# Training may enhance early childhood educators’ self-efficacy to lead physical activity in childcare

**DOI:** 10.1186/s12889-021-10400-z

**Published:** 2021-02-19

**Authors:** Brianne A. Bruijns, Andrew M. Johnson, Jennifer D. Irwin, Shauna M. Burke, Molly Driediger, Leigh M. Vanderloo, Patricia Tucker

**Affiliations:** 1 Health and Rehabilitation Sciences, Western University, London, Canada; 2 School of Health Studies, Faculty of Health Sciences, Western University, London, Ontario Canada; 3 School of Kinesiology, Faculty of Health Sciences, Western University, London, Ontario Canada; 4 Child Health and Evaluative Science, Hospital for Sick Children, Toronto, Ontario Canada; 5 School of Occupational Therapy, Faculty of Health Sciences, Western University, 1201 Western Road, Elborn College, Room 2547, London, ON N6G 1H1 Canada

**Keywords:** Early childhood educator, Childcare, Self-efficacy, Physical activity

## Abstract

**Background:**

Early childhood educators (ECEs) play a critical role in promoting physical activity (PA) among preschoolers in childcare; thus, PA-related training for ECEs is essential. The Supporting PA in the Childcare Environment (SPACE) intervention incorporated: 1. shorter, more frequent outdoor play sessions; 2. provision of portable play equipment; and, PA training for ECEs. An extension of the SPACE intervention (the SPACE-Extension) incorporated only the shorter, more frequent outdoor play periods component of the original SPACE intervention. The purpose of this study was to explore the individual impact of these interventions on ECEs’ PA-related self-efficacy and knowledge.

**Methods:**

ECEs from the SPACE (*n* = 83) and SPACE-Extension (*n* = 31) were administered surveys at all intervention time-points to assess: self-efficacy to engage preschoolers in PA (*n* = 6 items; scale 0 to 100); self-efficacy to implement the intervention (*n* = 6 items); and, knowledge of preschooler-specific PA and screen-viewing guidelines (*n* = 2 items). A linear mixed effects model was used to analyze the impact of each intervention on ECEs’ self-efficacy and knowledge and controlled for multiple comparison bias.

**Results:**

The SPACE intervention significantly impacted ECEs’ self-efficacy to engage preschoolers in PA for 180 min/day (main effect), and when outdoor playtime was not an option (interaction effect). Further, the interaction model for ECEs’ knowledge of the total PA guideline for preschoolers approached significance when compared to the main effects model. Participants within the SPACE-Extension did not demonstrate any significant changes in self-efficacy or knowledge variables.

**Conclusions:**

Findings from this study highlight the benefit of ECE training in PA with regard to fostering their PA-related self-efficacy and knowledge. Future research should explore the impact of PA training for ECEs uniquely in order to determine if this intervention component, alone, can produce meaningful changes in children’s PA behaviours at childcare.

**Supplementary Information:**

The online version contains supplementary material available at 10.1186/s12889-021-10400-z.

## Background

Physical activity (PA) is necessary to support young children’s (< 5 years) physical, cognitive, and psychosocial development [1]; while excessive sedentary time, particularly on screens, can slow young children’s cognitive and psychosocial development, and is associated with irregular sleep patterns [2]. Specific to centre-based childcare, preschoolers (3–5 years) engage in minimal moderate-to vigorous-intensity PA (MVPA; 4.6 min/hr) and spend the majority of their day sedentary (36.2 min/hr) [3]. Considering the large proportion of preschoolers (~ 65%) who spend close to 30 h/week in childcare [4, 5], there is great potential for the staff in this setting to model and shape movement behaviours, including PA and sedentary behaviour, which are known to be established in early childhood [6].

The childcare centre itself accounts for 50% of the variation in young children’s PA [7], stressing the importance of creating environments and shaping early childhood educator (ECE) behaviours in support of PA. Factors known to influence preschoolers’ PA in childcare include: environmental characteristics (e.g., portable play equipment, indoor play area, size/features of outdoor play area [8–10]; centre policies/practices (e.g., written policies for PA, provision of active/sedentary opportunities, scheduled outdoor time [9, 11]; and, ECE behaviours (e.g., prompts, co-participation, structured/unstructured play, staff training in PA) [8, 9]. While interventions in childcare have targeted environmental characteristics [12, 13] and centre policies [14, 15], an increasing focus has been placed on the influence of ECEs due to the prominent role they play in programming, leading, and modeling appropriate daily PA [16, 17]. In fact, for every additional 5 min/hour that ECEs engaged in MVPA, Carson et al. [18] found that children in their care engaged in an additional 1.3 min/hour of the same behaviour, stressing the influence ECEs can have on children’s behaviours. Additionally, with the large variation in environmental characteristics and policies among childcare centres [7], targeting ECEs may be necessary to mediate differences in PA rates attributable to centre-based characteristics.

Researchers have shown that programming active opportunities is often related to the value ECEs place on PA [16], as well as their self-efficacy to lead PA [19]. In fact, ECEs who reported low PA-related self-efficacy attributed this to their lack of training in PA [20]. According to Social Cognitive Theory, self-efficacy—defined as the confidence to complete a task—is one of the most robust determinants of behaviour [21]. As such, in order to better support ECEs’ effective facilitation of PA in childcare centres, fostering their self-efficacy in PA domains is important; one avenue to achieve this, suggested in the literature [22] and requested by ECEs themselves [23], is to provide ECEs with PA-specific training.

While the impact of PA training for ECEs has yet to be explored quantitatively in relation to their self-efficacy to promote and teach PA in childcare, the association of ECE training with PA rates of children has led to its incorporation in PA interventions in centre-based childcare [24, 25]. As a result of such PA training for ECEs, children have been found to engage in more MVPA [26, 27]. Specifically, a recent randomized controlled trial (RCT) incorporated PA training for ECEs [25], which increased preschoolers’ MVPA in childcare (+ 4 min/8-h day). In an extension of this RCT [25], results showed that ECEs (*n* = 17) reported feeling more knowledgeable and confident in their ability to lead PA opportunities following training and communicated that they would use the PA knowledge gained in future programming [28]. Given the noted increase in PA levels of preschoolers in this intervention [25], fostering ECEs’ knowledge and confidence in leading PA in childcare may have beneficial and lasting effects on the daily programming of active opportunities in this setting.

### The Supporting PA in the Childcare Environment (SPACE) study

In light of the influence of the childcare setting on young children’s PA and sedentary time [7, 9, 10], the Supporting PA in the Childcare Environment (SPACE) study, a 3-component intervention which aimed to increase preschoolers’ PA levels and decrease their sedentary time within centre-based childcare, was developed and implemented [29]. In short, the SPACE intervention incorporated: 1. a modified outdoor play schedule (i.e., shorter, more frequent outdoor play periods [four 30-min periods instead of the traditional two 60-min periods]); 2. ECE training in PA (i.e., one 4-h session covering topics including PA and sedentary behaviour guidelines for young children, how to facilitate PA in childcare, and overcoming barriers to PA, etc.); and, 3. environmental modifications (i.e., provision of portable play equipment [balls, hula hoops, etc.]).

Following the short-term success of the SPACE intervention [30], an extension of the SPACE study [31], using *only* the outdoor play schedule modifications (and no PA-related training for ECEs), was tested to see if this particular component of the intervention was responsible for the changes in movement behaviours observed in the original/initial SPACE trial [30].

### Objectives and hypotheses

This study explored the *individual* impact of the SPACE and SPACE-Extension interventions on ECEs’ PA-related self-efficacy and knowledge. It was hypothesized that ECEs in the experimental group of the original SPACE study (i.e., who received the PA training) would report an increase in their PA and implementation-related self-efficacy and knowledge relative to ECEs in the control group. In contrast, it was hypothesized that ECEs in the SPACE-Extension experimental group would not report an increase their PA-related self-efficacy and knowledge relative to the control group, as the SPACE-Extension intervention did not incorporate PA training for ECEs.

## Methods

This study presents results of two single-blind cluster-RCTs. Full methodological details of the SPACE study and SPACE-Extension study are reported previously [29, 31]. None of the data from the self-efficacy or knowledge variables reported in this manuscript have been reported previously.

### Participants and recruitment

For the initial SPACE study, ECEs of the preschool classroom(s) of 22 randomly selected eligible childcare centres (i.e., had ≥1 preschool classroom, ECEs and children were English-speaking) in London, Ontario agreed to participate and signed a written consent form. Using a blocked design, participating childcare centres were randomly assigned to the experimental or control group. Start dates for centres were staggered during the spring/summer of 2015 to allow feasible baseline data collection prior to randomization. All childcare centres participated for the full duration of the study. Only experimental condition centres received the SPACE intervention (i.e., were provided with portable play equipment, asked to implement four daily 30-min outdoor play sessions, and were provided with PA training for ECEs), while control condition centres continued their typical daily programming (i.e., two 60-min outdoor play periods). Ethical approval for the study was received from Western University’s Research Ethics Board (REB# 105779) and was registered with an International Standard Randomized Controlled Trial Number (ISRCTN 70604107).

The SPACE-Extension study followed the same protocol as the original SPACE study but recruited fewer centres (*n* = 12, none of which were in the original SPACE study) to pilot test one component of the original SPACE intervention (shorter, more frequent outdoor play sessions) during the spring/summer of 2017. Control centres continued with their traditional outdoor play schedule.

### Data collection

All assessments were completed by ECEs in both the experimental and control groups at baseline (i.e., week 0) and post-intervention (i.e., week 8) for both the SPACE and SPACE-Extension studies, and at 6- and 12-months post-intervention for the SPACE study only (given preschoolers’ PA returned to baseline levels in the SPACE study after the intervention ceased, 6- and 12-month follow-ups were not conducted in the SPACE-Extension study). Research staff (*n* = 2), blind to group allocation, visited centres prior to each data collection time point to distribute and collect questionnaires.

### Tools

The *Childcare Provider PA Self-Efficacy Questionnaire* (informed by Bandura’s *Guide for Constructing Self-Efficacy Scales* [32] and tested for face validity via expert consensus; *n* = 22 items; Additional File 1) was developed for this study to assess ECEs’ PA-related self-efficacy. For the purposes of this analysis, 12 of the self-efficacy items were used (6 pertaining to ECEs’ self-efficacy to engage preschoolers in PA, and 6 specific to ECEs’ self-efficacy to implement the SPACE and SPACE-Extension interventions. Implementation self-efficacy was only assessed in the experimental group. Self-efficacy items were scored on a scale from 0 (I am not at all confident) to 100 (I am completely confident), with a score of 50 representing a moderate level of confidence (as recommended by Bandura) [32].

The *Childcare Provider PA Questionnaire* (*n* = 23 items; Additional File 2) was derived from the validated short-form International PA Questionnaire [33], with additional questions created by the research team covering PA-related knowledge and awareness of centre policies. Within this survey, ECEs’ knowledge of the *Canadian PA and Sedentary Behaviour Guidelines for the Early Years* [34, 35] (*n* = 2 items) was assessed. A brief 6-item demographics questionnaire (i.e., sex, age, ethnicity, education level, employment status [full-time/part-time], and years of experience) was also completed by ECEs during baseline measurements in both the SPACE and SPACE-Extension studies.

### Data analysis

Descriptive statistics were analyzed in SPSS© (version 25) to report on ECE characteristics. All other statistical analyses were performed in R© [36], with linear mixed effects analyses conducted using the lme4 [37] and lmerTest [38] packages. Comparisons amongst time periods were assessed using the emmeans package [39]. To test the statistical significance of the prediction of each dependent variable by the fixed effects of interest, we adopted a hierarchical model testing strategy. We tested “main effects” models (models that did not allow group and time to interact) against “null models” (models in which the dependent variable was predicted by error) to determine the extent to which fixed effects produced significant change in dependent variables. For analyses in which we considered the interaction between time and group, the “interaction model” (models that allowed group and time to interact) was considered to be statistically significant if the interaction term significantly contributed to the prediction of a dependent variable, above and beyond either a significant main effects model, or the null model, in the event of a statistically non-significant main effects model.

Six discrete self-efficacy variables were analyzed to explore ECEs’ self-efficacy to engage preschoolers in PA, in both the original SPACE and the SPACE-Extension studies. Although it is possible that these variables were intercorrelated via an underlying “PA competence” variable, we evaluated these variables independently. To control for the possibility of multiple comparison bias, we evaluated the model for each dependent variable with an alpha of .05/6 = 0.0083. Post-hoc pairwise comparisons between intervention timepoints, where appropriate, were evaluated at an alpha of .05. To examine ECEs’ self-efficacy to engage preschoolers in PA, two linear mixed effects models were used, with group (experimental vs. control) and time (baseline, post-intervention, 6- and 12-month follow-up [SPACE], and baseline and post-intervention only [SPACE-Extension]) entered as fixed effects.

ECEs’ self-efficacy to carry out the SPACE and SPACE-Extension interventions was also explored through the use of a series of linear mixed-effects models. In these models, the dependent variable was predicted only by time, as these variables only pertained to ECEs in the experimental group. There were six discrete implementation self-efficacy variables, which were evaluated independently. As such, an alpha of 0.05/6 = 0.0083 was used to control for the possibility of multiple comparison bias.

Two variables examined ECEs’ knowledge of the PA and sedentary behaviour guidelines for preschoolers. ECEs who responded “180 min” for the PA guideline question (i.e., the minimum recommended amount of PA per day [34]) were scored as answering the question correctly, and ECEs giving the response of “60 min” for the sedentary behaviour guideline question (i.e., the maximum amount of screen time per day [35]) were scored as answering the question correctly. The proportion of correct responses by ECEs were explored using a linear mixed effects model, with group (experimental vs. control) and time (baseline, post-intervention, 6- and 12-month follow-up [SPACE], and baseline and post-intervention only [SPACE-Extension]) entered as fixed effects.

## Results

### Demographic information

#### SPACE

Eighty-three ECEs participated in the SPACE study (36.86 ± 10.40 years; 97.26% female). Most ECEs were Caucasian (86.96%), college-educated (69.23%), and worked full-time (95.16%, Table 1). No significant differences in baseline characteristics were found between ECEs in the control and experimental groups.

**Table 1:** *Early Childhood Educators’ Demographic Information*

Variable	***SPACE***		***p*** value	***SPACE-Extension***		***p*** value
	Control	Exp.		Control	***Exp.***	
**Age (years),** ***M*** **(*SD*)**	38.18 (12.73)	36.28 (9.45)	.62	34.00 (7.20)	36.20 (14.40)	.65
**Sex (male/female),** ***n***	0/27	2/44	.15	0/12	0/10	–
**Ethnicity**			.48			.84
Caucasian	21	39		11	9	
African Canadian	0	2		0	0	
Aboriginal	0	0		0	0	
Arab	0	1		0	0	
Latin-American	0	1		0	0	
Asian	2	3		1	0	
Other	0	0		0	1	
**Education**			.76			.16
High School	0	0		0	1	
College	8	19		9	17	
University	3	9		2	3	
Graduate School	0	0		1	0	
**Employment Status**			.08			–
Full-time	20	39		12	10	
Part-time	0	3		0	0	
**Years of Experience**			.90			.80
< 5	3	6		3	4	
5–9	2	8		2	1	
10–14	0	4		5	0	
15–19	4	1		0	2	
20+	2	9		2	2	

*Note. SPACE* Supporting Physical Activity in the Childcare Environment; *Exp.* Experimental; Frequencies (*n*) unless otherwise noted; Frequencies per item may not total *n* = 83 (SPACE) or *n* = 31 (SPACE-Extension) due to missing demographic data; Groups were compared using independent samples *t*-tests for continuous data, and *X*^*2*^ tests for categorical data; −- = *p* values could not be computed due to insufficient cell size

#### SPACE-Extension

Thirty-one ECEs participated in the SPACE-Extension study (35.00 ± 10.83 years; 100% female). Most were Caucasian (90.91%), college-educated (77.27%), and worked full-time (100%; Table 1). No significant differences were found in baseline characteristics between the control and experimental groups. See Table 1 for ECEs’ demographic information.

### Self-efficacy to engage preschoolers in PA

#### SPACE

**Fig. 1:**
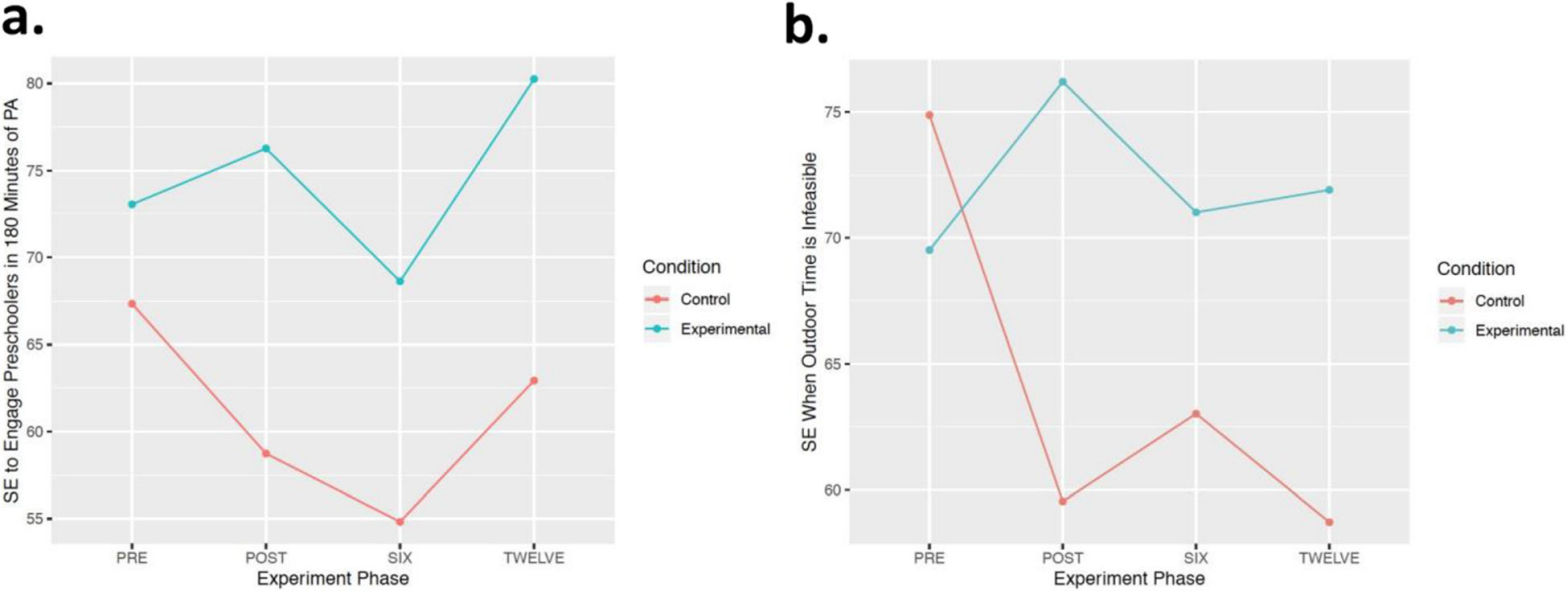
(**a**) Change in early childhood educators’ self-efficacy (SE) to engage preschoolers in 180 min of physical activity (PA) per day, at any intensity (*SPACE study*); (**b**) Change in early childhood educators’ SE to engage preschoolers in PA when outdoor time is infeasible (*SPACE study*)

There was a statistically significant difference between the main effects model and the null model for ‘engaging preschoolers in PA for 180 minutes each day, at any intensity’ (*X*^*2*^ [4] = 18.44, *p* = .001*; Table 2); however, after adjusting for multiple comparison bias, there was no effect of the intervention on this self-efficacy variable over time (Fig. 1a). There was, however, a statistically significant effect of the intervention for ‘engaging preschoolers in PA when outdoor playtime is not an option’ (*X*^*2*^ [7] = 19.90, *p* = .006*; Table 2, Fig. 1b).

**Table 2:** *Early Childhood Educators’ Self-Efficacy to Engage Preschoolers in Physical Activity*

***SPACE***										
***How confident are you that you can engage the preschool children in your care in physical activity …***	**Baseline**		**Post-Intervention**		**6-Months**		**12-Months**		**Interaction Effect**^**ϴ**^	
	**Control** ***M*** **(*SD*)**	**Exp** ***M*** **(*SD*)**	**Control** ***M*** **(*SD*)**	**Exp** ***M*** **(*SD*)**	**Control** ***M*** **(*SD*)**	**Exp** ***M*** **(*SD*)**	**Control** ***M*** **(*SD*)**	**Exp** ***M*** **(*SD*)**	***X***^**2**^	***p***^**a**^
In general (at any intensity)?	88.0 (12.7)	87.1 (15.4)	85.6 (14.8)	88.9 (13.7)	88.0 (15.4)	87.4 (13.2)	86.1 (14.2)	87.5 (16.0)	4.01	.779^b^
For 180 min each day (at any intensity)?	65.5 (25.6)	73.1 (18.1)	58.0 (25.5)	76.1 (17.4)	51.0 (30.2)	69.6 (22.3)	57.8 (30.0)	78.7 (15.7)	6.02	.11
At any intensity, for at least 30 min while *indoors*?	82.0 (22.0)	78.5 (20.7)	66.7 (25.7)	82.3 (14.0)	66.5 (24.3)	80.0 (21.5)	68.3 (29.8)	79.6 (18.0)	17.61	.014^b^
At any intensity, for at least 30 min while *outdoors*?	89.7 (15.5)	86.9 (16.5)	89.6 (13.7)	89.1 (15.1)	86.5 (14.6)	87.0 (16.4)	85.0 (17.2)	90.0 (13.5)	4.91	.671^b^
Outdoors when the weather is poor/unfavorable (e.g., cold, windy)?	71.7 (23.2)	75.1 (23.2)	62.2 (26.4)	76.3 (17.2)	65.5 (24.4)	74.1 (19.3)	66.7 (22.2)	76.9 (18.5)	11.34	.125^b^
When outdoor playtime is not an option (i.e., raining, heat alert, freezing weather conditions)?	74.5 (21.6)	69.6 (22.5)	59.6 (26.4)	77.2 (19.6)	62.5 (21.2)	71.9 (22.3)	58.9 (24.2)	72.2 (23.9)	19.90	.006^b,^*
***SPACE-Extension***										
***How confident are you that you can engage the preschool children in your care in physical activity …***	**Baseline**				**Post-Intervention**			**Interaction Effect**^**ϴ**^		
	**Control** ***M*** **(*SD*)**		**Exp** ***M*** **(*SD*)**		**Control** ***M*** **(*SD*)**		**Exp** ***M*** **(*SD*)**	***X***^**2**^		***p***^**a**^
In general (at any intensity)?	93.1 (9.5)		73.5 (22.7)		86.7 (14.1)		80.0 (18.9)	7.72		.052^b^
For 180 min each day (at any intensity)?	76.2 (14.5)		59.6 (25.4)		78.9 (14.5)		66.0 (21.0)	5.02		.170^b^
At any intensity, for at least 30 min while *indoors*?	86.2 (12.6)		48.1 (26.1)		80.0 (14.1)		62.7 (24.3)	3.44		.064
At any intensity, for at least 30 min while *outdoors*?	91.5 (12.1)		78.9 (22.2)		88.8 (12.5)		82.0 (17.8)	3.62		.306^b^
Outdoors when the weather is poor/unfavorable (e.g., cold, windy)?	83.9 (20.6)		58.9 (31.1)		75.0 (18.5)		64.0 (24.4)	5.55		.135^b^
When outdoor playtime is not an option (i.e., raining, heat alert, freezing weather conditions)?	82.3 (15.4)		55.0 (24.7)		76.3 (25.0)		58.7 (24.5)	0.49		.483

*Note*. *SPACE* Supporting Physical Activity in the Childcare Environment; *M* Mean self-efficacy rating (scale: 0 to 100); SD standard deviation; ^ϴ^The extent to which the interaction term added significantly to the prediction of the dependent variable; ^a^Adjusted alpha was set at 0.0083 to account for multiple comparison bias; *Significant intervention effect over time; ^b^Interaction model compared to null model, owing to statistically non-significant main effects model

#### SPACE-Extension

There was a significant difference between the main effects model and the null model for ECEs’ self-efficacy to engage preschoolers in PA ‘at any intensity, for at least 30 minutes while indoors’ (*X*^*2*^ [2] = 13.73, *p* = .001*; Table 2); however, the interaction model demonstrated no significant difference from the main effects model (*X*^*2*^ [3] = 3.44, *p* = .063; Table 2), suggesting no effect of the intervention over time.

### Self-efficacy to implement the intervention

#### SPACE

In general, ECEs in the experimental group (*n* = 49) reported high self-efficacy to implement the SPACE intervention (range = 72.8 to 85.6; Table 3). While the interaction model for this self-efficacy variable was statistically significant (*X*^*2*^ [3] = 13.38, *p* = .004), the only significant comparison was from post-intervention to 6-month follow-up (*t* [93] = 3.69, *p* = .002), where a decrease in self-efficacy was observed (from 85.6 to 72.8; Table 3). No other implementation self-efficacy variables significantly changed over time (*p* > .0083).

**Table 3:** *Experimental Group Early Childhood Educators’ Implementation Self-Efficacy*

***SPACE***						
***How confident are you that you can …***	**Baseline**	**Post-Intervention**	**6-Months**	**12-Months**	**Interaction Effect**^**ϴ**^	
	***M*** **(*SD*)**	***M*** **(*SD*)**	***M*** **(*SD*)**	***M*** **(*SD*)**	***X***^**2**^	***p***^**a**^
Implement the SPACE intervention?	80.7 (16.3)	85.6 (18.0)	72.8 (18.8)	80.0 (16.2)	13.38	.004*^b^
Modify the curriculum?	75.1 (23.4)	77.9 (26.1)	63.1 (29.2)	72.6 (22.6)	5.78	.123
Come up with a solution, if met with a barrier?	76.4 (18.7)	76.3 (20.9)	70.4 (22.5)	71.3 (21.2)	1.70	.637
Carry out the intervention when an unplanned change or interruption occurs?	73.5 (18.4)	74.7 (18.8)	65.8 (21.8)	67.4 (20.1)	4.69	.196
Carry out the intervention when met with resistance from preschoolers?	72.9 (19.5)	69.1 (23.0)	61.5 (23.6)	65.7 (27.6)	5.47	.141
Carry out the intervention when met with resistance from staff/colleagues?	74.8 (19.1)	72.8 (20.6)	59.6 (24.4)	63.5 (24.1)	9.64	.022
***SPACE-Extension***						
***How confident are you that you can …***	**Baseline**		**Post-Intervention**		**Interaction Effect**^**ϴ**^	
	***M*** **(*SD*)**		***M*** **(*SD*)**		***X***^**2**^	***p***^**a**^
Implement the SPACE-Extension intervention?	75.4 (19.8)		76.2 (19.4)		0.02	.884
Modify the curriculum?	71.5 (21.2)		63.1 (24.6)		1.59	.207
Come up with a solution, if met with a barrier?	72.3 (17.9)		61.5 (25.8)		2.74	.098
Carry out the intervention when an unplanned change or interruption occurs?	70.0 (20.0)		62.3 (24.2)		1.95	.163
Carry out the intervention when met with resistance from preschoolers?	69.2 (19.8)		63.2 (24.3)		0.91	.341
Carry out the intervention when met with resistance from staff/colleagues?	71.5 (17.7)		60.8 (24.0)		2.78	.096

*Note. SPACE* Supporting Physical Activity in the Childcare Environment; ^ϴ^The interaction effect, as compared to the main effect; *M* Mean self-efficacy rating (scale: 0 to 100); *SD* standard deviation; ^a^Adjusted alpha was set at 0.0083 to account for multiple comparison bias; *Significant intervention effect over time; ^b^ The only comparison that was statistically significant was from post-intervention to 6-months

#### SPACE-Extension

In general, ECEs in the experimental group (*n* = 17) reported moderate-to-high self-efficacy to implement the SPACE-Extension intervention (range = 60.8 to 76.2; Table 3). The intervention did not have a significant effect on ECEs’ implementation self-efficacy over time (*p* > .0083).

### Knowledge of PA and sedentary behaviour guidelines

#### SPACE

**Fig. 2:**
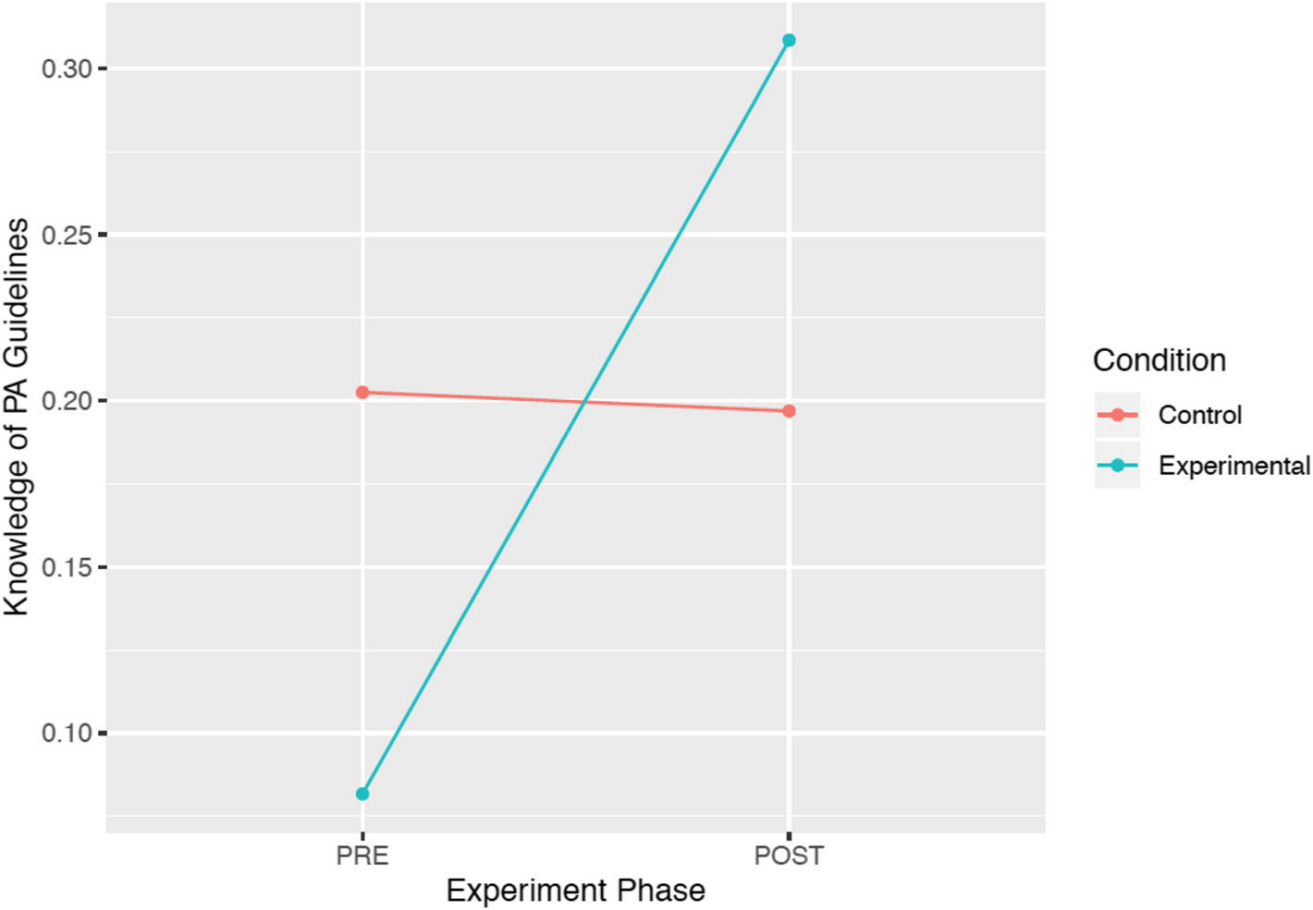
Change in early childhood educators’ knowledge of the total physical activity (PA) guideline for preschoolers (i.e., 180 min of total PA per day; Canadian Society for Exercise Physiology, 2012a; *SPACE study*)

In the PA guideline analysis, the interaction model was significantly better at predicting the dependent variable as compared with the null model (*X*^*2*^ [3] = 11.73, *p* = .008*), and approached significance when compared with the main effects model (*X*^*2*^ [1] = 4.91, *p* = .026). This suggests that the SPACE intervention may have benefitted ECEs’ knowledge of the PA guideline, over the course of the intervention (Table 4; Fig. 2). The interaction term was not statistically significant when considering ECE knowledge of the screen time recommendation (Table 4).

**Table 4:** *Early Childhood Educators’ Knowledge of Physical Activity and Sedentary Behavior Guidelines*

***SPACE***										
**Guideline**	**Baseline**		**Post-Intervention**		**6-Months**		**12-Months**		**Interaction Effect**^**ϴ**^	
	**Control** ***M*** **% (*SD*)**	**Exp** ***M*** **% (*SD*)**	**Control** ***M*** **% (*SD*)**	**Exp** ***M*** **% (*SD*)**	**Control** ***M*** **% (*SD*)**	**Exp** ***M*** **% (*SD*)**	**Control** ***M*** **% (*SD*)**	**Exp** ***M*** **% (*SD*)**	***X***^**2**^	***p***^***c***^
Total Physical Activity^a^	20 (41)	8 (28)	17 (38)	31 (47)	20 (41)	33 (48)	26 (45)	38 (50)	4.91	.026
Screen Time^b^	12 (33)	13 (34)	14 (35)	19 (40)	10 (31)	21 (42)	5 (22)	22 (42)	1.01	.799^d^
***SPACE-Extension***										
**Guideline**	**Baseline**				**Post-Intervention**				**Interaction Effect**^**ϴ**^	
	**Control** ***M*** **% (*SD*)**		**Exp** ***M*** **% (*SD*)**		**Control** ***M*** **% (*SD*)**		**Exp** ***M*** **% (*SD*)**		***X***^**2**^	***p***^***c***^
Total Physical Activity^a^	15 (38)		10 (32)		0 (0)		21 (43)		3.24	.357^d^
Screen Time^b^	8 (28)		0 (0)		17 (41)		7 (27)		1.55	.672^d^

*Note*. *SPACE* Supporting Physical Activity in the Childcare Environment; ^ϴ^The interaction effect, as compared to the main effect; *M %* Mean percent of educators correctly recalling the guideline; *SD* standard deviation; *Exp* experimental; ^a^180 minutes at any intensity per day (CSEP, 2012a); ^b^Should not exceed one hour per day (CSEP, 2012b); ^c^Adjusted alpha was set at 0.025 to account for multiple comparison bias; ^d^Interaction model compared to null model, owing to statistically non-significant main effects model; *Significant intervention effect over time

#### SPACE-Extension

The interaction term was not significant within the PA guideline analysis or within the screen time guideline analysis, suggesting that the SPACE-Extension intervention did not result in a change in ECEs’ knowledge of these guidelines for preschoolers (Table 4).

## Discussion

This study was the first to explore ECEs’ self-efficacy about PA for preschoolers and knowledge of PA and screen time guidelines following the implementation of PA interventions, with and without a PA training component, in centre-based childcare. The SPACE intervention positively impacted select items pertaining to ECEs’ self-efficacy to engage preschoolers in PA, while the SPACE-Extension intervention demonstrated no effect on ECEs’ self-efficacy. ECEs’ knowledge of both the PA and the screen time guidelines did not significantly change following either intervention; however, changes observed in the SPACE intervention approached significance for knowledge of the PA guideline. These results highlight important considerations regarding the inclusion of PA training for ECEs. Several important findings are discussed below.

ECEs’ self-efficacy to engage preschoolers in PA may be an important indicator of their related teaching behaviours in childcare [40, 41]. As such, fostering their self-efficacy through PA training remains an important avenue to explore. For example, in the SPACE study, ECEs in the experimental group (who received PA training) increased their self-efficacy to both engage preschoolers in 180 min of daily PA, and to facilitate PA opportunities when outdoor play was not feasible, compared to the control group. On the contrary, the only significant change in ECEs’ self-efficacy in the SPACE-Extension, where no staff training was offered, was a result of regression to the mean. These results were not surprising, as PA-related education has been found to positively affect self-efficacy in early childhood education students [40, 42, 43]. Altunsöz and colleagues [42] explored the effect of completing a physical education and games course on early childhood education students’ (*n* = 83) self-efficacy to teach fundamental movement skills (FMS) for preschoolers and found a significant main effect of the course on students’ self-efficacy to teach FMS (*p* = <.001). Similarly, Bruijns and colleagues [40] surveyed 1292 early childhood education students across Canada and found that those who had taken one or more PA-related courses reported higher self-efficacy for engaging young children in appropriate daily MVPA than those who had no training in PA. Taken together, these findings suggest that PA training may be highly beneficial for ECEs, who have communicated there is a gap in such training in their post-secondary program [40, 43]. While only a few self-efficacy items demonstrated a significant change as a function of the SPACE intervention, efforts to integrate PA education into ECEs’ pre-service programs and include more opportunities for professional development in PA in childcare professions may help to foster ECEs’ PA-related self-efficacy.

Equally as beneficial for ECEs as fostering their self-efficacy to engage preschoolers in PA is to ensure they have appropriate self-efficacy to implement health-promoting changes in childcare centres. Even though ECEs’ implementation self-efficacy remained relatively unchanged over the course of both interventions, they reported high initial implementation self-efficacy, which persisted even at the cessation of the intervention. This suggests that despite the burden of modifying the outdoor play curriculum and managing the new equipment, ECEs still felt confident in their ability to carry out the intervention. This finding is important, as PA interventions in centre-based childcare have been found to be most effective when led by ECEs [44]. As such, designing an intervention that is supported and easily administered by ECEs is essential. In a recent pilot study, ECEs (*n* = 11) were trained on how to break up bouts of sitting time (> 20 min) with various activities (e.g., active story time, movement breaks) [45]. The authors reported that ECEs exhibited high intervention fidelity and were also highly positive about the intervention, showing the benefit of providing training and support for ECEs when implementing movement behaviour interventions in childcare. In the present study, while no significant changes in implementation self-efficacy were observed, ECEs in the SPACE-Extension reported a slight decrease in their self-efficacy to carry out the intervention at post-intervention compared to their baseline scores, while ECEs’ implementation self-efficacy in the original SPACE study remained relatively constant. As such, while no concrete conclusions can be drawn from these changes, incorporating training for ECEs as an intervention component may ease its implementation and acceptability, which are both important factors for eventual adoption.

PA training for ECEs in childcare is not only important to foster self-efficacy to engage preschoolers in PA and implement PA interventions, but also to improve their knowledge of PA guidelines. In the SPACE study, where ECEs were educated about the *Canadian PA Guidelines for the Early Years* [34] (among other topics), there was an increase in the proportion of ECEs in the experimental group that indicated the correct response for preschoolers’ minimum daily total PA, compared to the control group. Contrarily, the SPACE-Extension did not have a significant impact on ECEs’ knowledge of this guideline. Considering the majority of young children spend many hours in childcare [4, 5], this setting is where children accumulate the majority of their weekday PA [46]. Thus, it is important for ECEs to be knowledgeable about the PA guidelines and modify their programming to ensure sufficient movement is incorporated throughout the day. Previously, ECEs (*n* = 20) reported that it was not their responsibility to facilitate PA in their childcare classroom, as they perceived the children in their care were already active enough [47]. However, in light of the findings from the present study showing that most ECEs were unsure of PA guidelines for young children, it is possible that ECEs’ perceptions of sufficient levels of activity may not be accurate. Therefore, ensuring ECEs are provided with this type of education, coupled with strategies on how to incorporate PA into programming, may equip them with the knowledge and tools necessary to ensure guidelines are met.

In contrast with ECEs’ increased awareness of the PA guidelines in the SPACE study, the intervention did not have the same effect with respect to their awareness of the screen time guideline for preschoolers. Despite this content being presented as part of the ECE training in the original SPACE study, perhaps ECEs were not as attentive to this information because their centres may already have limitations regarding screen use (as is the case in ~ 29% [*n* = 178] of Canadian childcare centres [48]). Further, the screen time guideline for preschoolers is only one component of the sedentary behaviour guidelines [35]. Given preschoolers’ reduction in sedentary time as a result of the SPACE intervention (− 2.13 min/hr) [30], ECEs may have been more interested in the content pertaining to reducing sitting time and breaking up long bouts of sedentary behaviour than the specifics regarding screen time limits. Nevertheless, Vanderloo [49] reported in her systematic review that young children engage in 0.1 to 1.3 h/day of screen-viewing while in centre-based childcare. As such, if screens are permitted in the centre, it remains important that ECEs are cognizant of the screen time guidelines, as well as the harmful effects of excessive screen use for young children [2].

### Research implications for public health

This study provides preliminary evidence that training ECEs in PA can increase their confidence to lead PA in childcare settings, even in the face of barriers such as inclement weather. Considering that preschoolers in the intervention group of the original SPACE trial increased their PA relative to those in the control group [30], it is plausible that this may have been, in conjunction with the outdoor play schedule and provision of portable play equipment, due to ECEs’ increased PA-related confidence and their knowledge of the PA guideline. However, given that only a select few of the self-efficacy findings reached significance, research employing a validated self-efficacy tool with a larger sample of ECEs is needed for a more robust evaluation of this construct. Despite these few significant findings, the potential public health impact of providing all ECEs with PA training is vast. Such professional learning opportunities for ECEs may change the way they value PA experiences for children in the childcare setting, thus increasing the likelihood that they will incorporate more active play opportunities in their daily programming. While recent research by Bruijns and colleagues [50] has highlighted PA and sedentary behaviour content that should be included in ECE training (e.g., topics such as outdoor play, benefits of PA in early childhood, and factors of the childcare setting that influence PA), further research is necessary in order to determine the appropriate duration of training and what is the optimal mode of delivery before scaling up PA training to be offered to all ECEs across Canada [51].

### Limitations

While this study reports the effects of two complementary clustered-RCTs on the self-efficacy and knowledge of ECEs with regard to preschooler PA, it is not without its limitations. First, ECEs hired in large childcare organizations (e.g., YMCA) often rotate among affiliated centres. Therefore, with the 2-year gap between interventions, it is possible that ECEs in the experimental group of the SPACE study (who received the training) may have moved to a new centre participating in the SPACE-Extension. While this introduces the possibility of cross-contamination, measures were taken to avoid this (ensuring different centres were recruited for the SPACE-Extension, refraining ECEs from completing questionnaires if they had already received the original SPACE intervention). Second, direct comparison of the two studies was not possible due to variation in the number of measurement timepoints, as well as the disparate nature of baseline scores among groups; therefore, we were unable to yield concrete conclusions as to whether it was the ECE training that benefitted ECEs’ PA-related self-efficacy and knowledge of the PA guidelines. Third, considering both the SPACE and SPACE-Extension interventions were conducted in childcare centres in London, Ontario exclusively, findings may not be generalizable to other jurisdictions.

## Conclusion

This study provides evidence that PA training for ECEs can positively influence their self-efficacy to engage preschoolers in PA, as well as increase their knowledge of PA guidelines. Taken together, these benefits are likely to have significant public health impacts; improved PA-related self-efficacy and knowledge of PA guidelines may translate to ECEs’ teaching behaviours, where knowledge of PA guidelines and self-efficacy to engage preschoolers in PA might serve to increase the quantity and quality of active play opportunities provided to young children in childcare. Future interventions in childcare should focus on professional development for ECEs to determine if tailored PA training can independently impact PA rates of preschoolers. Further, providing more support for ECEs in the form of increased training opportunities may help foster their competence and perceived capability to organize and design PA experiences for their children; such supports may lead to more intentional monitoring and programming of PA in childcare curricula.

## Supplementary Information

**Additional file 1.**

**Additional file 2.**

## Data Availability

The datasets generated and analyzed during the present study is available from the corresponding author upon reasonable request.

## Abbreviations

ECE: Early Childhood Educator
ISRCTN: International Standard Randomized Controlled Trial Number
MVPA: Moderate-to Vigorous-intensity Physical Activity
PA: Physical Activity
RCT: Randomized Controlled Trial
REB: Research Ethics Board
SPACE: Supporting Physical Activity in the Childcare Environment
