# Supplementary material for: Training may enhance early childhood educators’ self-efficacy to lead physical activity in childcare
Source: BMC Public Health. 2021 Feb 19;21:386. doi: 10.1186/s12889-021-10400-z (PMC7893737; doi:10.1186/s12889-021-10400-z)
Supplement: Supplementary file 1 — Additional file 1. [file 12889_2021_10400_MOESM1_ESM.pdf]

Participant ID: ____________

Supporting Physical Activity in the Childcare Environment: The SPACE Study

<u>Childcare Provider Self-Efficacy Questionnaire</u>

Researchers at the University of Western Ontario and McMaster University are undertaking one of the first Canadian studies aimed at creating a health promotion-based physical activity intervention for preschool-aged children attending centre-based childcare.

In answering the following questions you will be asked to think about how confident you are that you can participate in physical activities that are described as *light and moderate-to-vigorous* in intensity. Additionally, you will be asked to think about how confident you are that you can: a) engage the children in your care in physical activity; and, b) implement the SPACE physical activity intervention. The word “confident” refers to the belief that you have in yourself that you can do something well.

A. PERSONAL PHYSICAL ACTIVITY BEHAVIOURS

- **LIGHT ACTIVTIES:** Are when you are moving around, but your heart rate and breathing do not increase very much. You probably will not be sweating unless the weather is really hot. You would be able to talk easily through the activity.
- **MODERATE-TO-VIGOROUS ACTIVITIES:** Are when your breathing and heart rate increase (i.e., your heart will be beating fast and you will be breathing harder). You will likely be sweating. You may also find it hard to talk during the activity.

1. How confident are you that you can complete **30 minutes** of physical activity at a **light** intensity level <u>each day</u>?

**0**	10	20	30	40	**50**	60	70	80	90	**100**
I am not at all confident					*I am somewhat confident*					*I am completely confident*

2. How confident are you that you can complete **150 minutes** of physical activity at a **light** intensity this week?

**0**	10	20	30	40	**50**	60	70	80	90	**100**
I am not at all confident					*I am somewhat confident*					*I am completely confident*

3. How confident are you that you can complete **30 minutes** of physical activity at a **moderate-to-vigorous** intensity level each day?

**0**	10	20	30	40	**50**	60	70	80	90	**100**
I am not at all confident					*I am somewhat confident*					*I am completely confident*

4. How confident are you that you can complete **150 minutes** of physical activity at a **moderate-to-vigorous** intensity this week?

**0**	10	20	30	40	**50**	60	70	80	90	**100**
I am not at all confident					*I am somewhat confident*					*I am completely confident*

How confident are you that you will still be able to participate in 150 minutes of physical activity at a moderate-to-vigorous intensity per week, when faced with the following situations:

5. When the weather is bad?

**0**	10	20	30	40	**50**	60	70	80	90	**100**
I am not at all confident					*I am somewhat confident*					*I am completely confident*

6. When you are tired?

**0**	10	20	30	40	**50**	60	70	80	90	**100**
I am not at all confident					*I am somewhat confident*					*I am completely confident*

7. If you have other time commitments (e.g., family, friends, etc.)?

**0**	10	20	30	40	**50**	60	70	80	90	**100**
I am not at all confident					*I am somewhat confident*					*I am completely confident*

8. If you are busier than normal?

0 10 20 30 40 **50** 60 70 80 90 **100**

I am not at all confident *I am somewhat confident* *I am completely confident*

9. Are there any other things that might stop you from taking part in regular physical activity? If so, please describe below:

____

____

____

____

B. ENGAGING PRESCHOOLERS IN PHYSICAL ACTIVITY

1. In general, how confident are you that you can engage the preschool children in your care in physical activity (at any intensity)?

0 10 20 30 40 **50** 60 70 80 90 **100**

I am not at all confident *I am somewhat confident* *I am completely confident*

2. How confident are you that you can engage the preschool children in your care in physical activity for **180 minutes (i.e., 3 hours) each day** (at any intensity)?

0 10 20 30 40 **50** 60 70 80 90 **100**

I am not at all confident *I am somewhat confident* *I am completely confident*

3. How confident are you that you can engage the preschool children in your care in physical activity (at any intensity) for at least 30 minutes while *indoors*?

0 10 20 30 40 **50** 60 70 80 90 **100**

I am not at all confident *I am somewhat confident* *I am completely confident*

4. How confident are you that you can engage the preschool children in your care in physical activity (at any intensity) for at least 30 minutes while *outdoors*?

0 10 20 30 40 **50** 60 70 80 90 **100**

I am not at all confident *I am somewhat confident* *I am completely confident*

5. How confident are you that you can still engage the preschool children in your care in physical activity outdoors when the weather is poor/unfavourable (e.g., cold, windy)?

0 10 20 30 40 **50** 60 70 80 90 **100**

I am not at all confident *I am somewhat confident* *I am completely confident*

6. How confident are you that you can still engage the children in your care in physical activity when outdoor playtime is not an option (i.e., raining, heat alert/warning, freezing weather conditions)?

0 10 20 30 40 **50** 60 70 80 90 **100**

I am not at all confident *I am somewhat confident* *I am completely confident*

C. IMPLEMENTING THE INTERVENTION

The SPACE Intervention aims to improve the physical activity levels of preschool-aged children during childcare hours. More specifically, this intervention aims to increase this group's activity levels by (1) including additional portable play equipment, (2) introducing changes to daily programming/curriculum, and (3) providing physical-activity related training to staff.

Now having completed the SPACE program, we would like to know how confident you are with regards to the following items.

1. How confident are you that you can implement the SPACE physical activity intervention?

0 10 20 30 40 **50** 60 70 80 90 **100**

I am not at all confident *I am somewhat confident* *I am completely confident*

2. How confident are you that you can modify the environment (i.e., ensure that the portable play equipment is available) at your centre to better support the physical activity levels of the children in your care?

0 10 20 30 40 **50** 60 70 80 90 **100**

I am not at all *I am somewhat* *I am completely*

confident *confident* *confident*

3. How confident are you that you can modify the curriculum (i.e., re-structure outdoor play time to four 30-minute sessions/day) at your centre to better support the physical activity levels of the children in your care?

0 10 20 30 40 **50** 60 70 80 90 **100**

I am not at all confident *I am somewhat confident* *I am completely confident*

4. How confident are you that you can come up with a solution if met with a barrier to implementing and/or carrying out the intervention?

0 10 20 30 40 **50** 60 70 80 90 **100**

I am not at all confident *I am somewhat confident* *I am completely confident*

5. How confident are you that you can carry out the intervention when an unplanned change or interruption occurs in your daily schedule?

0 10 20 30 40 **50** 60 70 80 90 **100**

I am not at all confident *I am somewhat confident* *I am completely confident*

6. How confident are you that you can carry out the intervention when met with resistance from the children?

0 10 20 30 40 **50** 60 70 80 90 **100**

I am not at all confident *I am somewhat confident* *I am completely confident*

7. How confident are you that you can carry out the intervention when met with resistance from other staff members/colleagues?

0 10 20 30 40 **50** 60 70 80 90 **100**

I am not at all confident *I am somewhat confident* *I am completely confident*

Thank you for completing this questionnaire.
