# Supplementary material for: Training may enhance early childhood educators’ self-efficacy to lead physical activity in childcare
Source: BMC Public Health. 2021 Feb 19;21:386. doi: 10.1186/s12889-021-10400-z (PMC7893737; doi:10.1186/s12889-021-10400-z)
Supplement: Supplementary file 2 — Additional file 2. [file 12889_2021_10400_MOESM2_ESM.pdf]

Participant ID: ____________

Western
UNIVERSITY · CANADA

Supporting Physical Activity in the Childcare Environment: The SPACE Study

Childcare Provider Physical Activity Questionnaire

Researchers at the University of Western Ontario and McMaster University are undertaking one of the first Canadian studies aimed at creating a health promotion-based physical activity intervention for preschoolers attending centre-based childcare.

Please choose one response for each of the questions listed below. There are no 'right' or 'wrong' answers. Thank you!

A. PHSYICAL ACTIVITY-RELATED PRACTICES AND ENVIRONMENT

1. Does your childcare facility formally incorporate "energetic play" in the curriculum as part of outdoor play periods?

☐ Yes
☐ No

2. Does your childcare facility formally incorporate physical activity in the curriculum for indoor play?

☐ Yes
☐ No

3. Does your childcare facility incorporate structured physical activity (i.e., adult-led/adult-directed) in the curriculum?

☐ Yes
☐ No

4. Does your childcare facility incorporate unstructured physical activity (i.e., child-led/adult-directed) in the curriculum?

☐ Yes
☐ No

5. Is there sufficient space at your childcare facility for preschoolers to engage in physical activity indoors (e.g., adequate space in classroom, separate gross motor room/gym)?

☐ Yes
☐ No

6. Is there sufficient space at your childcare facility for preschoolers to engage in physical activity <u>outdoors</u>?

- ☐ Yes
- ☐ No

7. Does your childcare facility have any physical activity-related policies?

- ☐ Yes, please describe: ____
- ☐ No

8. How often do you discuss the importance of physical activity with the children in your classroom?

- ☐ Daily
- ☐ A couple times a week
- ☐ A couple times a month
- ☐ Rarely

9. Does your childcare facility have any screens (and related activities; e.g., TV, computers) available/accessible to preschool children?

- ☐ Yes, go to question 10
- ☐ No, go directly to question 12

10. If **YES**, which kinds?

- ☐ TV
- ☐ Computer
- ☐ Video games
- ☐ Other: ___________________________

11. Does your childcare facility have any screen-viewing related policies (for TV and computers)?

- ☐ Yes, please describe: ____
- ☐ No

12. Do you feel that you are a good physical activity role model for the children you care for?

- ☐ Yes, definitely
- ☐ Somewhat, but could be better
- ☐ Not sure
- ☐ No, not really

Please explain your answer.

B. PHYSICAL ACTIVITY AWARENESS

1. How physically active would you say the preschoolers **you care for** are?

- ☐ Inactive
- ☐ Somewhat active
- ☐ Very active

2. On average, how many minutes **per day** are the children in your care scheduled to engage in **physical activity** of *any* intensity indoors?

- ☐ Less than 30 minutes
- ☐ 30-59 minutes
- ☐ 60-89 minutes
- ☐ 90-119 minutes
- ☐ 120-149 minutes
- ☐ 150-179 minutes
- ☐ 180 minutes or more

Please describe the typical physical activities scheduled:

____
____
____
____

3. On average, how many minutes **per day** are the children in your care scheduled to engage in **physical activity** of *any* intensity outdoors?

- ☐ Less than 30 minutes
- ☐ 30-59 minutes
- ☐ 60-89 minutes
- ☐ 90-119 minutes
- ☐ 120-149 minutes
- ☐ 150-179 minutes
- ☐ 180 minutes or more

Please describe the typical physical activities scheduled:

____
____
____
____

4. If you are familiar with the Canadian **Physical Activity Guidelines for the Early Years**, do you recall the **minimum amount** of time that preschoolers should be **physically active** at *any* intensity each day?

- ☐ 30 minutes
- ☐ 60 minutes
- ☐ 90 minutes
- ☐ 120 minutes
- ☐ 180 minutes
- ☐ I don’t know how many minutes
- ☐ I’m not familiar with these guidelines

5. If you are familiar with the Canadian **Sedentary Behaviour Guidelines for the Early Years**, do you recall the amount of **screen time** preschoolers should be **limited to** per day?

- ☐ 30 minutes
- ☐ 60 minutes
- ☐ 90 minutes
- ☐ 120 minutes
- ☐ 180 minutes
- ☐ I don't know how many minutes
- ☐ I'm not familiar with these guidelines

C. YOUR PHYSICAL ACTIVITY LEVELS – THE INTERNATIONAL PHYSICAL ACTIVITY QUESTIONNAIRE (SHORT FORM)

1. How important is physical activity in your life?

- ☐ Not at all important
- ☐ Neither important or unimportant
- ☐ Somewhat important
- ☐ Very important

2. What is your main reason for engaging in physical activity?

- ☐ For health reasons
- ☐ For aesthetic reasons (i.e., physical appearances)
- ☐ To socialize with friends or family
- ☐ For pleasure
- ☐ Other: ____

The following questions are about the time you spent being physically active in the last 7 days. They include questions about activities you do at work, as part of your house and yard work, to get from place to place, and in your spare time for recreation, exercise or sport. Your answers are important.

Please answer each question even if you do not consider yourself to be an active person.

In answering the following questions:

- **Vigorous** physical activities refer to activities that take hard physical effort and make you breathe much harder than normal.
- **Moderate** activities refer to activities that take moderate physical effort and make you breathe somewhat harder than normal

3a. During the last 7 days, on how many days did you do **vigorous** physical activities like heavy lifting, digging, aerobics, or fast bicycling?

Think about *only* those physical activities that you did for at least 10 minutes at a time.

________ **days per week** ⇨ 3b. How much time in total did you typically spend on one of those days doing vigorous physical activities?

_______**hours** ___________**minutes**

OR

☐ **none**

4a. Again, think *only* about those physical activities that you did for at least 10 minutes at a time. During the last 7 days, on how many days did you do **moderate** physical activities like carrying light loads, bicycling at a regular pace, or playing doubles tennis? Do not include walking.

________ **days per week** ⇨ 4b. How much time in total did you typically spend on one of those days doing moderate physical activities?

_______**hours** ___________**minutes**

OR

☐ **none**

5a. During the last 7 days, on how many days did you **walk** for at least 10 minutes at a time? This includes walking at work and at home, walking to travel from place to place, and any other walking that you did solely for recreation, sport, exercise or leisure.

________ **days per week** ⇨ 5b. How much time in total did you typically spend walking on one of those days?

_______**hours** ___________**minutes**

OR

☐ **none**

The last question is about the time you spent sitting on weekdays while at work, at home, while doing course work and during leisure time. This includes time spent sitting at a desk, visiting friends, reading, traveling on a bus, or sitting or lying down to watch television.

6. During the last 7 days, how much time in total did you typically spend *sitting* on a **week day?**

________ **hours** ______ **minutes**

Thank you for completing this questionnaire.
